# Social Distancing in the Era of COVID-19: A Call for Maintaining Social Support for the Maternal Population

**DOI:** 10.9745/GHSP-D-20-00398

**Published:** 2021-06-30

**Authors:** Alaa Alhomaizi, Dalal Alhomaizi, Sandra Willis, Helen Verdeli

**Affiliations:** a Teachers College, Columbia University, New York, NY, USA.

## Abstract

In the era of COVID-19, pregnant and postpartum women, an already vulnerable group, are facing unforeseen and compounding stressful events with reduced social protections. We argue that to prevent harmful consequences that may surpass the effects of the crisis itself for pregnant women and their families, it is imperative to prioritize maintaining formal and informal sources of social support for mothers in proposed infection control policies.

## Key Messages

- Pregnant, laboring, and postpartum women are navigating the challenges inherent to the perinatal period against the backdrop of a global pandemic but without a key protective factor—social support.
- Formal and informal social support systems for mothers need to be prioritized, even during pandemics, and failure to do so will greatly affect mothers, their infants, and their whole households.
- When determining policies to mitigate the spread of coronavirus disease (COVID-19), policy makers should take a harm reduction approach that incorporates feasible and innovative strategies to ensure the continuation of maternal social support.
- Policy makers need to engage and empower mothers as well as the associated professional communities to voice their needs and to inform and participate in the policy formulation process to ensure the creation of policies that are better suited to maternal social support needs during the pandemic.

## INTRODUCTION

Over the past year, the strategies used around the globe to slow the spread of coronavirus disease (COVID-19) have radically changed everyday life for millions of households. Conversing behind masks, avoiding physical proximity with people from outside the household, and viewing one another as potential biohazard carriers have severely disrupted the social and cultural connections that are vital for human communities, especially during crises. The adverse mental health effects of previous infectious disease outbreaks, such as Ebola, severe acute respiratory syndrome (SARS), and H1N1 influenza epidemics, have been documented on a number of populations and subgroups.^1^^,^^2^ However, with the notable exception of HIV/AIDS, the literature on the mental health effects of recent epidemics has left out one particularly vulnerable group—expectant and new mothers.^3^^,^^4^ The current pandemic was different in this regard. There has been an increased awareness of its maternal mental health ramifications, with more than 2 dozen studies already published within a year on the mental health effects of the pandemic on pregnant and postpartum women.^5^^,^^6^ The majority of the studies highlighted the impact of containment measures implemented to stem the spread of COVID-19, such as social distancing, as core reasons for the increased maternal mental morbidity. Social distancing measures range from isolation of infected individuals, to closures of schools and nonessential businesses, to national lockdowns.^7^ As the world marks 1 year since the World Health Organization declared COVID-19 a global pandemic, nearly every country remains under some form of social distancing.

Since the beginning of 2020, pregnant, laboring, and postpartum women have been navigating the challenges inherent to the perinatal period in the context of sudden, severe, and cumulative stressful events with concurrent reductions of vital social connections and protections. It is therefore critical to mitigate the enormous impact of COVID-19 on maternal mental health. In this article, we argue that formal and informal social support systems for mothers need to be prioritized, even during pandemics, and failure to do so will greatly affect the mothers, their infants, and their whole households. Furthermore, we call for the dissemination of feasible and innovative adaptive strategies that ensure continuation of social support to this population.

## THE PERINATAL PERIOD: A VULNERABLE TIME

The perinatal period, which includes both the gestational and postpartum phases, is characterized by substantial biological changes and major life adjustments that can result in various degrees of emotional distress.^8^^,^^9^ Globally, a significant portion of perinatal women develop symptoms of depression and anxiety. In high-income countries (HICs), the prevalence of depression ranges from 7.0% to 20.0% for pregnant women and 9.3% to 13.0% for postpartum women.^10^^–^^13^ In a review of low- and middle-income countries (LMICs), higher prevalence rates of perinatal depression were generally found, with studies reporting prevalence rates that ranged from 4.8% to 57.0% and 3.8% to 48.5% for antepartum and postpartum depression, respectively.^14^ Although perinatal anxiety is not as widely investigated and understood as perinatal depression, research indicates that it is an equally common condition globally. The range of prevalence rates for antepartum anxiety is 5.8% to 35.9% in HICs compared to 6.6% to 61.1% in LMICs, while the prevalence rates of postpartum anxiety range from 4.7% to 36.1% in HICs compared with 2.2% to 38.3% in LMICs.^15^^–^^19^ Additionally, extensive literature over the past 50 years has shown associations between maternal mental illness and maternal morbidity and mortality, including suicide.^20^^,^^21^ Moreover, maternal antepartum and postpartum psychopathology has been associated with a range of adverse fetal (e.g., preterm delivery), child (e.g., emotional difficulties, externalizing behavior), and adolescent outcomes (e.g., increased risk for depression).^22^ These adverse effects are compounded by socioeconomic disadvantages, and in LMICs, they include worse child physical development outcomes, such as stunting.^23^

Many of the determinants of maternal psychopathology are exacerbated by the realities of life during the COVID-19 pandemic.^24^ Perinatal women must now cope with elevated stress in their environments due to heightened fears of infection and contamination, concern for their family's and their own health, and exposure to media coverage of the pandemic.^25^^,^^26^ Further, daily life has changed drastically due to increased isolation and loneliness as well as disruption of meaningful life experiences such as family gatherings, birth celebrations, and mourning rituals for the death of loved ones. Women and their families are also facing significant economic hardships as unemployment has surged and economies are struggling worldwide. Food shortages have been widely reported during the pandemic, increasing food insecurity, a known risk factor for maternal psychopathology.^27^ These experiences exacerbate the psychological toll of pregnancy, labor, and childcare. This circumstance is especially true for women with a history of mental disorders, who are at a heightened risk of developing episodes following exposure to stressful life events.^8^

Many of the determinants of maternal psychopathology are exacerbated by the realities of life during the COVID-19 pandemic.

Furthermore, the preexisting gender disparity in mental disorders will likely worsen as a result of the differential, deleterious impact of the pandemic on women in general and mothers in particular.^28^ Women have historically been significantly more likely than men to be living in poverty, doing considerably more domestic and unpaid work, and providing the majority of care to children, the elderly, and the sick.^29^ As COVID-19 swept the globe and lockdowns were instituted, this gender imbalance increased significantly, and women, particularly working mothers, have been taking on even more responsibilities at home to the detriment of their mental health.^29^^,^^30^ Although staying home protects people from infection, it can become at times an unsafe option for women. Countries have reported increases in domestic violence that range from 25% to 30% as communities went into lockdown.^31^^–^^33^ Critically, the lack of access to protective familial, community, and governmental networks due to social distancing measures may leave women without the social protections needed to flee a dangerous situation, hold their perpetrators accountable, or access the means for them and their children to survive after leaving their homes.^34^ This situation is a human rights and health catastrophe since intimate partner violence, historically higher during the perinatal period, is associated with a range of adverse obstetric, maternal, and child outcomes.^35^

Given the increase in the number and the impact of the determinants of maternal mental ill health, it is not surprising to see a subsequent rise in the prevalence rates of maternal psychopathology. In a systematic and meta-analytic review of maternal mental health studies during the COVID-19 pandemic, Yan and colleagues^5^ reported an upsurge in the prevalence rates of antepartum depression (8%–71%) and anxiety (11%–82%), as well as postpartum depression (15%–29%), surpassing prepandemic prevalence rates. Notably, the rates of psychopathology reported for the maternal population exceeded those of the general population and even the highly overburdened health care workers.^5^^,^^36^ Given the documented increases in the prevalence rates of maternal mental disorders and the heightened vulnerability of this population, it is vital to harness protections to mitigate the effects of the pandemic on pregnant and postpartum women.

## LIFE DURING A PANDEMIC: THE PERFECT STORM OF HIGH STRESS AND LOW SUPPORT

A well-established protective factor against maternal mental morbidity is social support,^37^ defined as the comfort and assistance that persons receive from members of their social network “to help them cope with biological, psychological, and social stressors.”^38^ There are various forms of social support that are beneficial to mothers, including emotional, informational, and instrumental support.^37^ Social support is associated with lower rates of antepartum and postpartum depression and increased maternal self-efficacy.^39^^–^^41^ It also mitigates the effects of maternal depression on child outcomes, decreasing behavioral problems and the overall risk of mental disorders in children.^42^ Of critical importance, social support has a “buffering” effect on stressors. In fact, the more stressful a situation is, the more impactful social support will be.^37^

However, as people navigate the “new normal” of living with social and movement restrictions, countries and community members are deciding what is considered “essential” and what can be forgone to ensure infection control. Frequently, that has meant restricting physical proximity at the expense of social connection. As a result, community support systems, from maternal support groups to breastfeeding and prenatal classes, and informal in-person support systems, such as visits from friends and relatives, are significantly less available to perinatal women. Even before the pandemic, aspects of motherhood could be lonely and isolating, especially for first-time mothers.^43^^,^^44^ These social networks were sources of aid, encouragement, and community that served as a lifeline for many women. However, during the pandemic, mothers are finding themselves without support when they most need it.

During the pandemic, mothers are finding themselves without support when they most need it.

Moreover, women have to routinely engage with health care facilities before, during, and after childbirth. But, due to infection control measures instituted to reduce the spread of COVID-19 in health care settings, perinatal women are interacting with a medical system that lacks many of the support networks that existed prepandemic. A number of health care facilities globally, including many in HICs, have reduced prenatal visits and prohibited support persons, such as partners, from being present during prenatal appointments.^45^^,^^46^ During labor and delivery, many hospitals are banning doulas and support persons from attending births. After birth, some hospitals are limiting in-person lactation consultations, forbidding visitations from family and friends, and restricting parents and other helpers from visiting neonatal intensive care units.^47^^,^^48^ And, in LMICs, where mothers frequently report unsupportive birthing experiences in health facilities, including disrespectful, neglectful, and abusive treatment from providers, their birth experience may worsen due to the pandemic-related burden on the health care systems.^49^

## CHILDCARE DURING COVID-19: DIMINISHED SUPPORT NETWORKS

Beyond their pregnancy, laboring, and birthing experience, numerous mothers will be spending their postpartum period in a radically restricted society in which they will have to limit their interactions to members of their own households. Globally, there are cultural social support practices that risk disruption, such as the postpartum recovery rituals found in many regions that require at-home support from female relatives and caregivers to ensure rest and recuperation for the new mother.^50^ These practices have been consistently shown to provide instrumental, emotional, and informational support for the mother and to reduce the risk of postpartum depression.^51^ Moreover, social support can help mothers engage in activities that promote mental well-being, such as exercise and spending time outdoors.^52^

Worldwide, many mothers rely on grandmothers to help with childcare, which has been shown to protect against parenting stress and maternal depression as well as improve child outcomes.^41^^,^^53^^,^^54^ However, many mothers are losing this vital source of support because of restricted visitation between households owing to social distancing mandates, especially those for older adults. In LMICs, where multigenerational households are typical, live-in grandparents are a source of much-needed support and reprieve for the mothers. However, live-in elders face an increased risk of infection when they share their home with family members who leave the house for work or food aid. Also, since they are more vulnerable to the effects of the infection, they disproportionately become sick or perish, leaving the household members bereaved and helpless. Therefore, the tension between benefits and costs associated with social support for mothers has become enormous during this pandemic.

The tension between benefits and costs associated with social support for mothers has become enormous during this pandemic.

In both high- and low-income regions, partner support is the most predictive of maternal mental and physical health outcomes, surpassing every other type of social support.^8^^,^^55^^–^^58^ In every stage of the perinatal journey, from pregnancy to postpartum, the partner's involvement is central to maternal well-being and crucial to stress alleviation.^58^^,^^59^ Intimate partners are a vital source of emotional support, offering affection, reassurance, and encouragement to women as they navigate the challenges of motherhood. They also provide instrumental support, including practical, financial, and most importantly, childcare support. With diminished help from outside the household due to COVID-19 restrictions, women need their partner's aid more than ever. Yet, numerous countries reported that despite a substantial increase in fathers' contributions to household duties during the pandemic, mothers were still shouldering most of the increased burden of childcare and housework.^60^^–^^62^

Expectant and new working mothers have been experiencing substantial levels of psychological distress and morbidity during the pandemic, with a study revealing that as much as a third of pregnant working women are experiencing significant anxiety.^5^ Global studies have revealed that the disruption of childcare support within the home (i.e., domestic caregiver) as well as outside of the home (i.e., childcare centers) during the pandemic has had a disproportionate impact on working mothers.^60^^,^^63^^,^^64^ For instance, compared with working fathers, working mothers are 3 times as likely to be the only household member providing childcare.^64^ Moreover, historically, whenever childcare has been unavailable or unaffordable, it is usually mothers who end up choosing to stay home, work part time, or quit their jobs.^65^^–^^67^ This pattern also seems to be the case during the current pandemic.^68^ Furthermore, worldwide, women are 1.8 times as likely to lose their job due to COVID-19 as men, which increases the risk for maternal mental distress and psychopathology.^69^ Therefore, the pandemic-induced childcare crisis and its resultant impact on maternal employment is one of the biggest threats to maternal well-being.

Among mothers struggling with lost childcare support, there are particularly vulnerable populations. For example, in LMICs, 64% to 90% of women work in the informal sector and do not have the economic stability to afford to not work to care for their infants.^70^ In the past, this frequently meant that children were left home without adult supervision or that the mother brought her children to work with her,^54^ both options that are especially dangerous during the coronavirus pandemic. The mental burden of working in unsafe conditions with little formal protections, coupled with the ambivalence of either leaving one's children unattended or exposing them to the pandemic's hazardous conditions, may put these women at an especially high risk for significant distress. For another vulnerable group, single mothers in both HICs and LMICs, who tend to rely on support from their family or community, social distancing rules may result in enormous isolation and lack of support.^71^ Pandemic guidelines that do not account for the social support needs of perinatal women will have disproportionate psychological consequences on these especially vulnerable women.

Pandemic guidelines that do not account for the social support needs of perinatal women will have disproportionate psychological consequences on these women.

## CONSIDERATIONS FOR POLICY MAKERS

Many experts believe that COVID-19 and its effects will be around for some time and that even with the approval of multiple safe and effective vaccines, the pandemic will not end abruptly.^72^^,^^73^ Further, in LMICs, widespread availability of vaccines may not be possible until 2023.^74^ At the beginning of this crisis, a core mitigation strategy was for everyone to stay at home and everything that was deemed not essential be closed or banned.^75^ However, a public health strategy that may be appropriate for an acute crisis that will resolve in the short term may not be realistic or feasible for a crisis that will continue for some time. Now, a year into this pandemic^76^:

*people are seeking social contact not out of selfishness but because, like going to the grocery store, human connection is an essential activity.*

During the COVID-19 pandemic, nationwide public health guidelines, such as 24-hour lockdowns, that did not consider the needs of the maternal population, led to catastrophic consequences, including maternal deaths and stillbirths.^77^^,^^78^ These incidents suggest a dangerous parallel to prior epidemics in which maternal and neonatal deaths indirectly caused by the epidemic surpassed the number of deaths directly caused by the infection.^79^ Therefore, a need exists to alter COVID-19–related policies and practices of health and social care systems to become more inclusive of the needs of mothers, including their increased need for social support. Policy makers need to engage and empower mothers as well as the associated professional communities to voice their needs, inform, and participate in the policy formulation process. Participatory policy-making practices increase transparency and accountability, and will help ensure the creation of policies that are better informed of maternal needs during the pandemic.^80^

Further, we recommend that policies implement a “harm reduction” approach to social interactions rather than the “abstinence-only” approach that was initially adopted during the pandemic.^81^ Rather than perceiving risk as all or nothing, a harm reduction approach to public health policies is based on the understanding that risk is not binary and that the risk of action should be compared against its benefits as well as the risk of inaction.^81^^,^^82^ The transmission dynamics of COVID-19 and their multiple impacts on people, while not fully known, are better understood now than at the start of the pandemic.^83^ As a result, infection control guidelines, such as recommending that everyone wear masks, have developed to reflect the latest empirical evidence. Similarly, policies and programs that impact the provision of social support for perinatal women need to be continuously informed by and consistently updated to reflect the newest research findings.

A harm reduction approach to public health policies is based on the understanding that risk is not binary and that the risk of an action should be compared against its benefits.

The following key considerations may guide policies that aim to be inclusive of maternal social support needs.

### Prioritize Safe Access to Support Services

- Ensure that any social support activity that could be done outdoors (e.g., support groups; and breastfeeding, prenatal, and parenting classes) is not banned given that the risk of COVID-19 transmission outdoors is significantly lower than indoors.^84^ These activities should include infection prevention strategies (e.g., social distancing, routine disinfecting, mask-wearing, and respiratory hygiene).
- Ensure perinatal women have access to remote mental health and social support services (e.g., support groups, psychological/psychiatric services, and counseling for gender-based violence), which may be via internet or mobile technology—the latter being much more common in LMICs.^85^^–^^87^
- Ensure active outreach is available, because many mothers may be reluctant, unable, or unwilling to reach out for help. For example, midwives, community health workers, and women's protection officers around the world have stepped in during this pandemic to reach out to mothers and ensure their well-being.^88^^,^^89^ Outreach may be done via mobile and internet technologies or safe in-person meetings.
- Offer safe options of in-person support for pregnant and postpartum women, including at-home for women who are quarantining at home to reduce their own or their newborn's risk of contracting COVID-19, especially if they or their infants are in an at-risk category (e.g., chronic lung disease, diabetes, cancer, or cardiovascular disease). For in-person services, the health and safety of the providers (i.e., community health workers; midwives; mental health counselors; and perinatal health care providers, such as doulas and breastfeeding consultants) must be prioritized as they provide this highly beneficial support to mothers.^90^^,^^91^ Safety protocols should include access to vaccines (if available), routine COVID-19 testing, and personal protective equipment for the support providers.

### Support the Safe Continuation of Childcare and Schooling

- Prioritize safe ways for childcare services to remain open^92^^,^^93^ or propose innovative alternatives for specific populations, such as infants and older children of essential workers or expectant and new mothers.^94^
- Provide safe options for children to receive their education in person, especially when community COVID-19 transmission rates are low. Infection prevention strategies that can be implemented to ensure a safe school environment may include ensuring access to COVID-19 testing and vaccines, as well as access to personal protective equipment for school staff; requiring physical distancing between individuals; performing routine disinfection; mandating mask wearing; encouraging respiratory hygiene; reducing school hours; reducing classroom sizes; using outdoor classrooms; and alternating attendance days for groups of students.
- Prioritize governmental investment in supported isolation and quarantine,^95^ such as providing alternative living arrangements for infected individuals living in crowded or multigenerational homes. Multigenerational homes are vital sources of support for new mothers, and safeguarding these environments is crucial for the physical and mental health of the whole family.

Policies and programs that impact providing social support for perinatal women need to be consistently updated to reflect the newest research findings.

### Ensure Positive Health Care Interactions and Birth Experience

- Commit to having a safe environment for childbirth to reduce potential harm on mother and infant due to direct and indirect effects of COVID-19, which includes ensuring that laboring women get tested for COVID-19 when they are admitted to the hospital and have personal protective equipment during their hospital stay.
- Safeguard a mother's right to a “positive birth experience,”^96^ which includes being “assisted by a kind and technically competent health care provider.” Support provided by health care providers is very beneficial to laboring women, and this is especially important because of the restrictions on other types of intrapartum support for laboring women due to COVID-19 restrictions.^97^
- Prioritize the safe presence of a support person during health care visits throughout the perinatal period, especially during labor, given its established importance for positive maternal outcomes.^87^ This includes ensuring that the support person has access to COVID-19 testing and personal protective equipment during the delivery to safeguard the mother, infant, support person, and health care workers.

## CONCLUSION

The coronavirus pandemic has shed light on the irrefutable mental health repercussions of global crises. Crucially, this pandemic highlighted the detrimental effects of overlooking vulnerable populations when developing crisis mitigation strategies. Therefore, as we continue to navigate the current emergency, it is vital to ensure that mothers and their families are able to receive the highest level of support safely possible as they manage these severe adversities. It is imperative that we prioritize the maternal population in COVID-19 policies, regulations, and recommendations to prevent harmful consequences that may surpass the effects of the crisis itself. Moreover, as we look on to the future after the pandemic, we ought to “build back better,” integrating the lessons we learned and innovations we discovered to ensure that we are better prepared for the next crisis. Given climate change, increased state fragility leading to the highest rates ever of forcibly displaced persons (the majority of whom are women and children), and the documented rise in infectious diseases, the lessons learned during this pandemic are critical for the protection of expectant and new mothers.

## Notes

**Peer Reviewed**

**First published online:** April 23, 2021.
